# HPLC-UV and GC-MS Methods for Determination of Chlorambucil and Valproic Acid in Plasma for Further Exploring a New Combined Therapy of Chronic Lymphocytic Leukemia

**DOI:** 10.3390/molecules26102903

**Published:** 2021-05-13

**Authors:** Katarzyna Lipska, Anna Gumieniczek, Rafał Pietraś, Agata A. Filip

**Affiliations:** 1 Department of Medicinal Chemistry, Medical University of Lublin, 20-090 Lublin, Poland; katarzyna.lipska@gmail.com (K.L.); rafal.pietras@umlub.pl (R.P.); 2 Department of Cancer Genetics with Cytogenetics Laboratory, Medical University of Lublin, 20-080 Lublin, Poland; a.filip@umlub.pl

**Keywords:** chlorambucil and valproic acid, HPLC-UV and GC-MS methods, optimization and validation, determination in plasma, combined anticancer therapy

## Abstract

High performance liquid chromatography with ultra-violet detection (HPLC-UV) and gas chromatography–mass spectrometry (GC-MS) methods were developed and validated for the determination of chlorambucil (CLB) and valproic acid (VPA) in plasma, as a part of experiments on their anticancer activity in chronic lymphocytic leukemia (CLL). CLB was extracted from 250 µL of plasma with methanol, using simple protein precipitation and filtration. Chromatography was carried out on a LiChrospher 100 RP-18 end-capped column using a mobile phase consisting of acetonitrile, water and formic acid, and detection at 258 nm. The lowest limit of detection LLOQ was found to be 0.075 μg/mL, showing sufficient sensitivity in relation to therapeutic concentrations of CLB in plasma. The accuracy was from 94.13% to 101.12%, while the intra- and inter-batch precision was ≤9.46%. For quantitation of VPA, a sensitive GC-MS method was developed involving simple pre-column esterification with methanol and extraction with hexane. Chromatography was achieved on an HP-5MSUI column and monitored by MS with an electron impact ionization and selective ion monitoring mode. Using 250 µL of plasma, the LLOQ was found to be 0.075 μg/mL. The accuracy was from 94.96% to 109.12%, while the intra- and inter-batch precision was ≤6.69%. Thus, both methods fulfilled the requirements of FDA guidelines for the determination of drugs in biological materials.

## 1. Introduction

**Figure 1:**
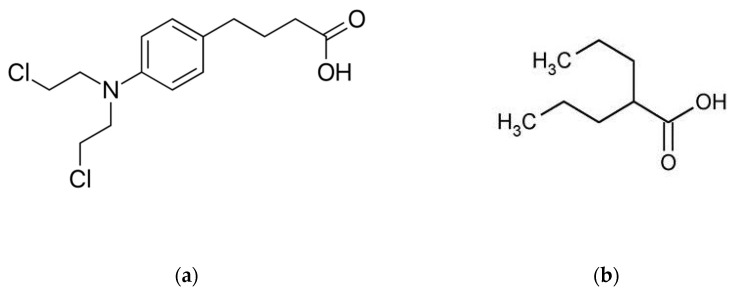
Chemical structures of (**a**) chlorambucil (CLB) and (**b**) valproic acid (VPA).

Chlorambucil (CLB) is a nitrogen mustard agent, chemically known as 4-(4-(bis(2-chloroethyl)amino)phenyl)butanoic acid (Figure 1). For many decades, CLB has been used as a standard therapy for patients with chronic lymphocytic leukemia (CLL). It acts as a bifunctional alkylating compound and possesses an ability to bind to different cellular structures by adding alkyls to a variety of electronegative groups, e.g., DNA bases. Its main anticancer activity is shown to be through cross-linking of DNA and inhibition of DNA replication [1,2]. It results in DNA fragmentation and wrong mRNA transcription from the damaged DNA. As a consequence, the cancer cells are unable to further divide [3]. CLB when administered orally is easily absorbed through the gastrointestinal tract and enters cells by simple diffusion [2]. However, the optimal dose of CLB and duration of treatment have not been established so far, despite the presence of CLB as a therapy for over 50 years [4]. Accordingly, its therapeutic concentration range in plasma is difficult to conclude and should be evaluated in a wide spectrum. In a study of 12 patients administered 0.2 mg/kg of CLB orally, a mean plasma concentration of 492 ± 160 ng/mL was measured [5].

A review of the literature on the quantitative determination of CLB in biological materials revealed only a few methods from the 1980s to 1990s, i.e., liquid chromatographic methods with ultraviolet detection (HPLC-UV) [6,7,8] and one liquid chromatography–tandem mass spectrometry (LC-MS/MS) method [9]. In recent years, LC-MS/MS methods were elaborated to determine CLB in human and animal plasma, and successfully applied to pharmacokinetic studies [10,11].

Valproic acid (VPA), a chemical molecule composed of two propyl groups combined with an acetic acid moiety (Figure 1), can be classified into the group of short-chain fatty acids [12]. It is a well known anticonvulsant drug which nowadays is widely examined for new therapeutic indications [13]. Analytical methods, such as HPLC [14,15,16,17,18], LC-MS/MS [19,20,21,22,23], gas chromatography (GC) [24,25,26] and gas chromatography–mass spectrometry (GC-MS) [27] were reported for the determination of VPA in biological matrices. Most of the previously reported HPLC and GC methods required complex sample pretreatment or detection, e.g., multistep derivatization for UV detection [14,17], multistep derivatization for fluorimetric detection [15,16], or complex extraction [18,24,25,26,27]. 

Due to the results from many experimental studies showing the ability of VPA to inhibit proliferation of different cancer cells, it was proposed as a candidate for the therapy of various types of cancer. Its antitumor activity could be based on inhibiting specific enzymes, i.e., histone deacetylases, up-regulating the expression of some types of G protein-coupled receptors and affecting Notch signaling activity [28]. What is more, it could potentiate the anticancer activity of many classic cytostatic drugs used in conventional anticancer therapy [13]. That is why the present study was designed as part of a large project embracing in vitro experiments in which the impact of CLB and VPA on the viability and apoptosis of cells isolated from the blood of patients with CLL was examined. The obtained results suggested synergistic anticancer effects of CLB and VPA that might lead to the development of a new therapy for CLL [29]. Thus, the main goal of the present study was to develop, optimize, and validate the methods for quantitative measurement of CLB and VPA in biological samples for further pharmacological, and pharmacokinetic studies.

## 2. Results and Discussion

The main goal of the present study was to develop reliable quantitative methods for the determination of CLB and VPA in plasma, as a part of our large project focused on the potent anticancer activity of CLB and VPA in combination. From the literature survey and our preliminary experiments, it became clear that it would not be possible to develop a simple and sensitive LC-UV or GC-UV method for the simultaneous determination of these drugs. It was because VPA is a compound without a strong chromophore that does not allow detection by conventional UV spectrometry above 210 nm (Figure S1). Previously used HPLC methods required many steps for derivatization, or other sophisticated procedures. In other methods, 2-bromo-4´-nitroacetophenone or 2-bromo-2´-acetonaphtone and 18-crown-6 ether were needed [14,17]. Before this, many steps, i.e., liquid-liquid extraction, centrifugation, separation and evaporation were required. Other authors proposed precolumn derivatization with a non-commercially available reagent, i.e., nOePeS [15] or 2-(2-naphthoxy)ethyl 2-(piperidino)ethanesulfonate reagent [16], which both needed fluorimetric detection. In addition, one previously published LC-MS/MS method was based on a two step derivatization with 2-chloro-1-methylpyridinium and 4-dimethylaminobenzylamine, shaking for 1 h and using evaporation [23].

Bearing in mind the high volatility of VPA, the GC-MS method seemed to be a suitable approach to increase the sensitivity of the assay. Thus, it was taken into account and used for the determination of VPA and CLB. Unfortunately, these preliminary GC-MS experiments showed extremely low volatility of CLB and a limit of quantification above 5 µg/mL (Figure S2). Since we needed a simple and relatively cheap procedure, and having available the HPLC-UV and GC-MS apparatus in our laboratory, we decided to develop two simple independent methods, i.e., HPLC-UV for CLB, and GC-MS for VPA. As a result, the presented methods were developed, optimized and validated according to international guidelines [30,31], and finally applied for the determination of CLB and VPA in plasma, at concentrations adequate for further pharmacological and pharmacokinetic experiments.

### 2.1. Optimization of HPLC Conditions for CLB Assay

**Figure 2:**
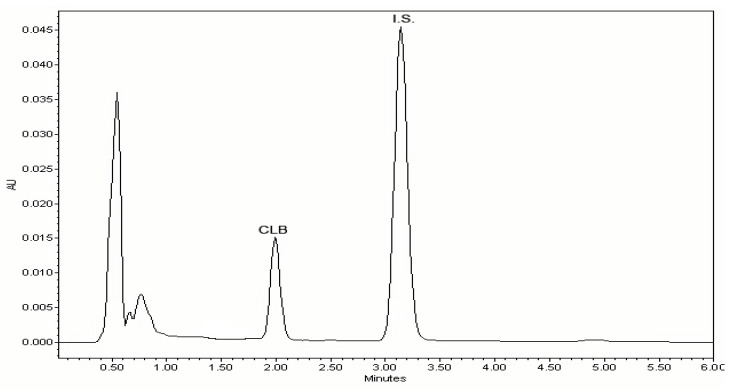
Representative chromatogram of plasma spiked with CLB at a concentration of 7.5 µg/mL; CLB and internal standard (I.S.) represent chlorambucil and mefenamic acid, respectively.

The chromatographic conditions were optimized over various experiments (different organic modifiers, different pH of the mobile phase, different columns) to achieve a short run time of analysis and symmetrical peaks for CLB and mefenamic acid (internal standard (I.S.)). It was found that the RP18 end-capped column and a simple mobile phase containing acetonitrile, water and formic acid at pH 2.6 were sufficiently effective for separation the analytes in a short time of below 3.5 min, as well as for the reduction of the peak tailing (Figure 2). At the same time, it was confirmed that VPA did not interfere with the assay of CLB (Figure S3).

Six solutions containing CLB and I.S. at a concentration of 15 µg/mL were injected onto the column for estimation of the system suitability. Retention times, resolution and the peak area ratios (CLB to I.S.) were evaluated according to the obtained results. Finally, we obtained the relative standard deviation (RSD) of peak area ratios below 4.0%, the RSD of the retention times (both CLB and I.S.) below 1.0%, and resolution between the analytes above 4 (Table 1), confirming that the system suitability parameters were met [31].

What is more, the presented CLB retention time was much shorter than that of 12 min which was reported previously [7]. Mefenamic acid was selected as an optimal I.S., based on the peak shape and suitable retention time under the chromatographic conditions described above, as well as on its extraction efficiency from plasma. Using the proposed method, the centrifuged and filtered layers from deproteinized plasma samples were analyzed without evaporation and concentration steps. As a result, the time and steps required for sample preparation were visibly reduced.

### 2.2. Selectivity

**Figure 3:**
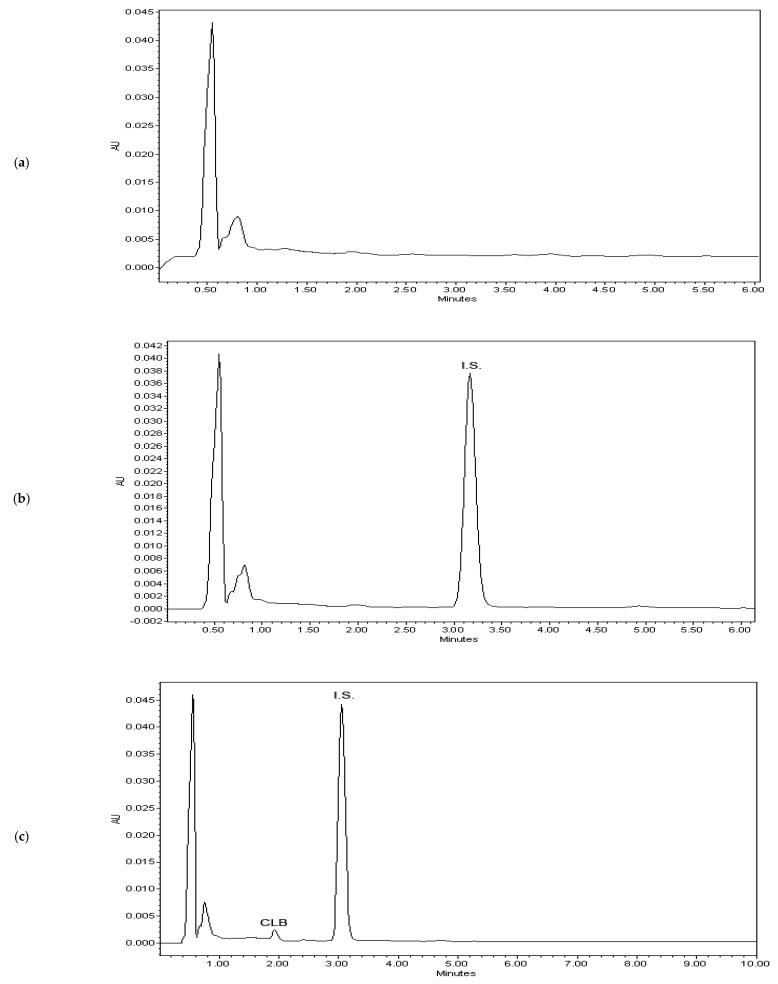
Representative chromatograms of (**a**) blank plasma, (**b**) zero sample with I.S. and (**c**) the lower limit of quantification (LLOQ) (0.075 µg/mL) for CLB.

The selectivity of the method was shown as the responses of the blank plasma (Figure 3a), zero plasma containing I.S. (Figure 3b) and the plasma sample containing CLB at the lower limit of quantification (LLOQ) level and I.S. (Figure 3c). These representative chromatograms indicated convincingly that no significant interferences from endogenous substances around the retention times of CLB and I.S. were observed.

### 2.3. Calibration and Sensitivity

The calibration curves showed linearity over the concentration range of 0.075–15 µg/mL with the mean correlation coefficient (r^2^) equal to 0.9996. The weighed (1/x) linear regression equation y = (0.15501 ± 0.00123)x + (0.00244 ± 0.00141) was obtained (mean ± SD), where y was the peak area ratio of CLB to I.S. and x was the concentration of CLB in µg/mL. 

The sensitivity of the method was determined as the lowest concentration of the standard samples within the range of quantification, with a signal-to-noise ratio of at least 10:1 (LLOQ), with an acceptable precision of less than 20%, and accuracy of 80–120% [30]. For the Quality Control (QC) samples at the LLOQ level, the mean intra-batch precision and accuracy were 9.09% and 94.13%, while the mean inter-batch values were 9.82% and 95.33%, respectively. All of these results were obtained using five replicated samples (Table 2).

While solid phase extraction (SPE) was used for the determination of CLB by the LC-MS/MS method, a limit of CLB quantification of 4 ng/mL was reported using 200 µL of plasma [9]. In turn, the HPLC-UV method offered an LLOQ of 30 ng/mL, but showed a much longer chromatographic run time (above 12 min) [7]. According to the literature, the mean plasma concentrations of CLB measured in plasma of patients after typical oral dosing were 492 ± 160 ng/mL [5]. Thus, the LLOQ proposed in the present study, which is equal to 75 ng/mL using 250 µL of plasma and a 10 µL of injection, could be acceptable for the determination of CLB in biological samples.

### 2.4. Precision and Accuracy

The acceptance criteria for the intra-batch and inter-batch precision, set at 15% for all QC samples, were achieved [30]. The intra-batch accuracy ranged from 94.13% to 101.12% with a precision (coefficient of variation, CV) of ≤9.46%. In addition, the inter-batch accuracy ranged from 95.06% to 97.60% with a precision of ≤9.82% (Table 2). These results indicated that our HPLC-UV method was sufficiently reproducible and accurate for quantification of CLB in plasma. Bearing in mind the results reported in the literature, i.e., the inter-day precision equal to 9.11% [10], the present method demonstrated a visibly comparable precision.

### 2.5. Recovery and Matrix Effect

The accuracy, expressed as recovery of CLB extracted from plasma in relation to nominal values, was in the range of 95.21–101.12% (Table 3). Thus, the mean recovery of the present method was higher than the 89% reported previously [9]. What is more, accurate and repeatable results were obtained using simple deproteinization with methanol followed by simple filtration. The present results also confirmed that the matrix components in plasma did not significantly affect the analytical responses. The matrix effect was evaluated by comparing the peak area ratios of CLB and I.S. after extraction from plasma, with the peak area ratios of the compounds analyzed directly. As a result, the levels from 95.45% to 100.33% were obtained for low, medium, and high concentrations of CLB (Table 3). Thus, it was suggested that significant matrix effects were not seen in the present method.

### 2.6. Stability

The stability of CLB was determined as the percentage ratio of the measured CLB concentration to its initial concentration in two QC samples, i.e., 0.15 µg/mL and 15 µg/mL. The results from the short-term, long-term and freeze-thaw stability tests are presented in Table 4. According to official guidelines the samples were considered stable when concentrations were within ± 15% of the nominal concentrations, and the precision was less than 15% [30]. CLB was stable in plasma at 23 °C for 24 h without visible degradation and with recovery in the range of 97.05–98.13%. CLB was also sufficiently stable in the long-term conditions with recovery in the range of 93.53–95.62%, and after three freeze-thaw cycles with recovery in the range of 93.67–94.99%. Furthermore, CLB was stable in the processed samples at 23 °C for 6 h with recovery ranging from 92.52% to 95.73%. In addition, the stock solutions for CLB and I.S. were stable for at least 3 weeks when stored at 4 °C.

### 2.7. Carryover Test

A carryover test was conducted by injecting a blank sample after injecting the sample with the highest concentration of CLB in the standard curve (15 µg/mL). The acceptance criterion of the carryover was that the peak in the blank sample should be less than 20% of the peak in the LLOQ concentration [30]. As a result, no peak of CLB above 20% was shown in the blank sample. Thus, it was confirmed that the carryover had no effect on further analysis of CLB.

### 2.8. Optimization of GC-MS Method for the Determination of VPA

An important aspect of the present study was that a relevant internal standard was used, tracking VPA as a main analyte during derivatization and extraction steps, and compensating for the possible matrix effect. Benzoic acid was found to be the best option for all these purposes. Similarly to VPA, it belongs to a group of carboxylic acids, but possesses a sufficiently distinct fragmentation pattern that allowed us to obtain clean MS chromatograms without significant interferences in the selective ion monitoring (SIM) channel. To optimize our experiments, a simple derivatization and extraction method was chosen from the literature [32] and optimized for both VPA and I.S. After that, the optimum conditions were established, the compounds were mixed together, derivatized and extracted, in order to perform their simultaneous determination.

We realize that precolumn derivatization of the analytes can be considered as a difficulty by many researchers. However, it was shown to be an essential step in the presented method, visibly reducing the peak tailing and increasing sensitivity. What is more, the simple esterification with methanol followed by the simple extraction with hexane occurred sufficiently fast, and was reproducible and effective. In addition, our procedure was very cheap, not requiring any sophisticated reagents, and allowing sufficient sensitivity.

**Figure 4:**
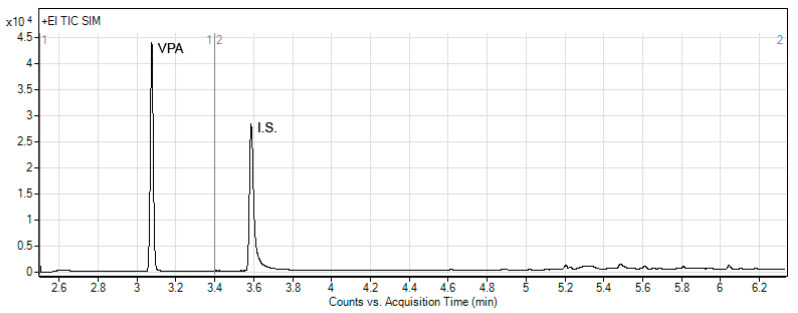
Representative chromatogram of plasma spiked with VPA at concentration of 7.5 µg/mL; VPA and I.S. represent valproic acid and benzoic acid, respectively.

During the development of the method, the GC and MS parameters were optimized as well. The optimal choice of the SIM ions was based on the ion mass spectra of VPA and benzoic acid (I.S.). It provided three SIM ions for VPA (*m*/*z* 159, 116, and 87) and three ions for benzoic acid (*m*/*z* 136, 105 and 77), which were applied for quantitative determinations. It was found that the optimized GC-MS procedure in the SIM mode clarified the chromatograms and provided the single peaks of VPA and I.S. (Figure 4).

### 2.9. Selectivity

**Figure 5:**
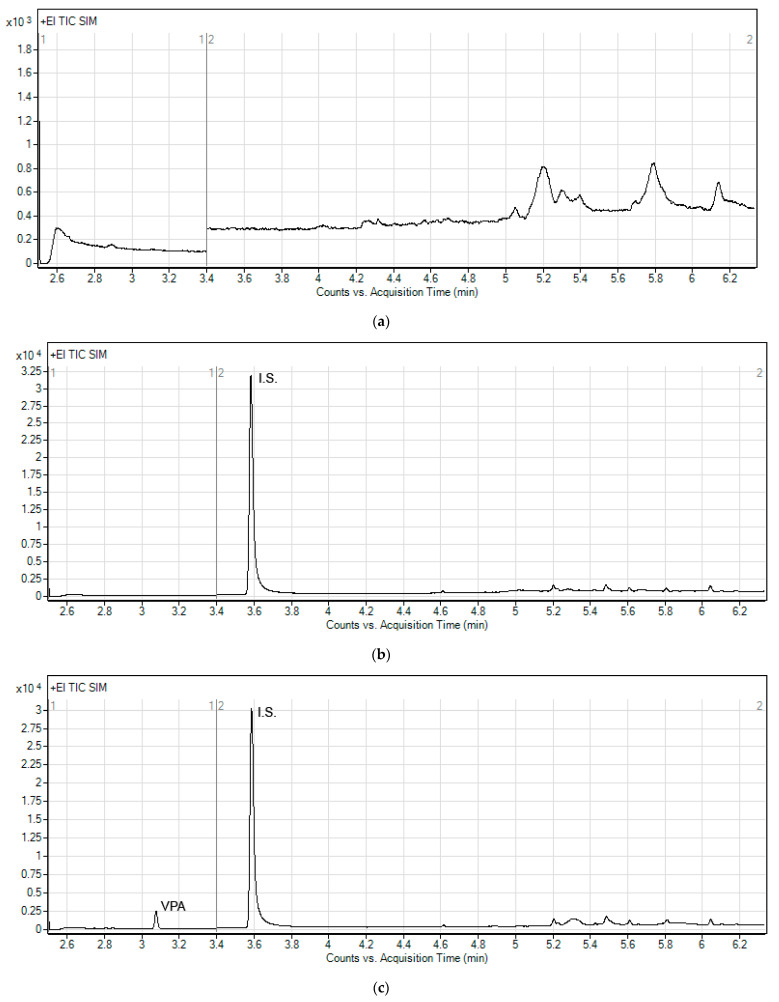
Representative chromatograms of (**a**) blank plasma, (**b**) zero sample with I.S. and (**c**) lower limit of quantification (0.075 µg/mL) for VPA. The selected ion monitoring (SIM) mode was used to monitor two subsets of fragments with their related mass values, the first (1) for VPA in the range 0–3.4 min, and the second (2) starting from 3.4 min for the internal standard (benzoic acid).

The selectivity of the method was shown as the responses of (a) the blank plasma, (b) zero plasma containing I.S. and (c) the plasma sample containing VPA at an LLOQ level and I.S. Representative chromatograms demonstrating no significant interferences from endogenous substances around the acquisition times of the analytes were shown in Figure 5. At the same time, it was confirmed that CLB spiked with respective plasma samples did not interfere with the determination of VPA (Figure S2).

### 2.10. Calibration and Sensitivity

The calibration curves were obtained by plotting the ratios of peak areas (VPA to I.S.) versus concentrations of VPA, and were shown to be linear over the concentration range of 0.075–15.0 µg/mL, with the mean determination coefficients (r2) equal to 0.9987 (*n* = 6). The weighed (1/x) linear regression equation y = (0.32003 ± 0.01209)x − (0.02029 ± 0.00279) (mean ± SD) was obtained, where y was the peak area ratio of VPA to I.S. and x was the concentration of VPA in µg/mL (Table 5).

The sensitivity of the method (LLOQ) was determined as the lowest concentration of VPA, within the range of linearity, with a signal-to-noise ratio of at least 10:1, with an acceptable precision of less than 20%, and accuracy of 80–120% [30]. For the QC samples at the LLOQ level, the mean intra-batch precision and accuracy were 5.78% and 94.13%, while the mean inter-batch values were 6.69% and 94.96%, respectively (Table 6).

One previously described HPLC method, based on the extraction of VPA with hexane from acidified plasma, evaporation and derivatization with 2-bromo-2´-acetonaphtone, offered the LLOQ of 0.05 µg/mL using 160 µL of plasma [14]. When LC-MS/MS methods were applied, the LLOQ of 0.2–5 µg/mL using 200 µL of plasma were reported [20,21,23]. In one of these methods, MS/MS detection required the extraction of VPA from acidified plasma with methylene chloride, then two step derivatization with 2-chloro-1-methylpyridinium iodide and 4-dimethylaminobenzylamine dihydrochloride, shaking for 1 h and using evaporation [23]. In turn, the centrifugal ultrafiltration followed by a direct GC method offered the linearity range from 0.56 µg/mL [24]. Because the mean concentration of VPA in plasma of patients with epilepsy is usually between 40 and 120 μg/mL [14,20,21], these LLOQ are optimal for the determination of VPA in patients with epilepsy after typical dosing. However, effective VPA dosing in different types of cancer is yet to be discovered. On the one hand, it is expected to be similar to that in epilepsy [33]. On the other hand, it could be lower, in order to reduce the side effects like somnolence and disorientation [34]. Thus, we were focused on developing a method which could be as sensitive as possible, allowing the determination of VPA when the typical therapeutic levels of VPA were not yet achieved. As a result, we obtained a linearity range of 0.075–15 µg/mL, with the required precision and accuracy. A similar linearity range of 50–5000 ng/mL was proposed recently in the literature, using a GC-MS/MS method and the Quick, Easy, Cheap, Effective, Rugged and Safe (QuEChERS) extraction [27].

### 2.11. Precision and Accuracy

Precision and accuracy were determined at four concentrations of VPA (LLOQ, QC 0.15 µg/mL, QC 3 µg/mL and QC 15.0 µg/mL) on two different days, and the acceptance criteria for the intra-batch and inter-batch precision were achieved [30]. The intra-batch accuracy ranged from 94.13% to 109.12% with a precision of ≤5.78%. In addition, the inter-batch accuracy ranged from 94.96% to 108.09% with a precision of ≤6.69% (Table 6).

### 2.12. Recovery and Matrix Effect

The accuracy, expressed as recovery of VPA after derivatization and extraction from plasma in relation to the nominal values, was in the range 96.07–106.61%, proving the method was adequately reliable and reproducible within the required analytical range. The matrix effect was evaluated by comparing the concentrations of VPA after extraction from plasma with those analyzed directly from methanol. The obtained results of 98.39–101.31% suggested that significant matrix effects were not seen in the present method. All these results are summarized in Table 7.

### 2.13. Carryover

A carryover test was conducted by injecting a blank sample after injecting the sample with the highest concentration of VPA in the standard curve (15.0 µg/mL). As a result, no peak of VPA more than 20% [30] of the peak at LLOQ concentration (0.075 µg/mL) was shown in the blank sample. Thus, it was confirmed that the carryover had no effect on further analysis of VPA.

### 2.14. Stability

The stability of VPA was determined as the percentage ratio of the measured VPA concentration to the initial VPA concentration in two QC samples, i.e., QC 0.15 µg/mL and QC 15.0 µg/mL. The results from stability studies are shown in Table 8. VPA was stable in plasma at 23 °C for 24 h without visible degradation, with recovery in the range of 95.06–96.87% and RSD in the range of 3.33–4.61%. VPA was also sufficiently stable in the long-term conditions with recovery in the range of 93.01–94.32% and precision in the range of 3.11–7.96%, and after three freeze-thaw cycles with recovery in the range of 86.86–96.41% and precision in the range of 2.55–8.64%. Furthermore, VPA was stable in the processed samples at 23 °C for 6 h with recovery ranging from 92.53% to 95.73%. Thus, all these ranges were within the limits recommended in the official guidelines [30]. In addition, the stock solutions for VPA and I.S. were stable for at least 3 weeks when stored at 4 °C.

Finally, we could conclude that the elaborated GC-MS method with simple derivatization ensured the quantitative determination of VPA, with desired sensitivity, precision and accuracy. Consequently, it could be a good alternative to previously described LC-MS methods [20,21,23,24] when used for monitoring VPA levels in biological samples.

## 3. Materials and Methods

### 3.1. Chemicals and Apparatus

Pure substances, CLB, VPA, mefenamic acid and benzoic acid, were purchased from Sigma-Aldrich (St. Louis, MO, USA). Human pooled plasma (HCV, HIV, HBsAG free) was purchased from MP Biomedicals Inc. (Aurora, OH, USA). Acetonitrile, methanol, hexane and formic acid for HPLC and GC-MS were obtained from Sigma-Aldrich or Merck KGaA (Darmstadt, Germany). Sulfuric acid 98% and sodium bicarbonate for analysis were obtained from POCh (Gliwice, Poland). Deionized water was produced in our laboratory with a Simplicity UV Water Purification System from Merck-Millipore (Burlington, MA, USA). A model of 375 centrifuge from MPW Med. Instruments (Warsaw, Poland), a drying oven SL115 from Pol-Eko Aparatura Sp. J. (Wodzisław Śląski, Poland), and a pH-meter HI9024C from Hanna Instruments (Villafranca Padovana, PD, Italy) were used. Nylon membrane filters (0.45 µm) and hydrophilic PTFE syringe filters (0.2 µm) from Merck were also applied.

### 3.2. Statistical Analysis

Statistica version 13.3 from TIBCO Software Inc. (Palo Alto, CA, USA) and free GNU R computational environment version 3.4.0 were used for the statistical analysis.

### 3.3. Validation of the Methods

The elaborated methods were validated according to the international regulations recommended by the Food and Drug Administration Agency [30,31]. Validation was performed by assessing selectivity, linearity, sensitivity, precision and accuracy, recovery and matrix effect, carryover and stability.

### 3.4. Selectivity

The selectivity of the methods was shown as the response of (a) the blank plasma samples without both drugs and respective I.S., (b) zero plasma samples without drugs but containing respective I.S., and (c) the plasma samples at the LLOQ concentration of CLB or VPA and I.S.

### 3.5. Linearity Estimation

Six point calibration curves were constructed by plotting the peak area ratios of CLB or VPA to respective I.S. (y) versus CLB or VPA concentrations (x). The slope, intercept and correlation coefficient r2 were calculated by weighted linear regression (1/x).

### 3.6. LOD and LLOQ

The limits of detection (LOD) were determined from the SD of the intercept and the slope of the mean regression lines. The lowest limits of quantification (LLOQ) were determined by analyzing progressively lower concentrations of CLB or VPA under the procedures described below, using a serial dilution method. The obtained LLOQ values were based on the signal-to-noise ratio of 10:1 which was determined by visual inspection.

### 3.7. Stability Tests

The stability of all stock solutions was examined after storage for 3 weeks at 4 °C. In addition, stability of CLB and VPA in plasma was assessed for short-term, long-term and freeze-thaw storages. Two different QC samples at low and high concentrations, i.e., 0.15 µg/mL and 15 µg/mL of CLB or VPA, were used for all stability tests. The samples were analyzed five times for every storage condition. The short-term stability test was conducted by maintaining the QC samples at 23 °C for 24 h, while the long-term stability was determined by freezing QC samples at −20 °C for 2 weeks. The QC samples were also stored at −20 °C for 24 h and then thawed at 23 °C. These steps were repeated three times for estimation of the freeze-thaw stability. In addition, the processed QC samples after extraction from plasma were checked after being placed on the table (CLB) or in an autosampler (VPA) at 23 °C for 6 h, for estimation of the post-preparative stability.

### 3.8. Carryover Tests

Carryover tests were conducted by injecting blank samples without the drugs after injecting the samples with the highest concentration of CLB or VPA in their calibration ranges (15 µg/mL).

### 3.9. Chromatographic Conditions for CLB Assay

The HPLC-UV method was performed using a model 515 pump, a Rheodyne 10 µL injector and a model UV 2487 detector, controlled by Empower 3 software, all from Waters UK (Elstree, Herts, England). Separation was performed on a LiChrospher® 100 RP18 end-capped column (125 × 4.0 mm, 5 µm particle size) from Merck. The mobile phase was a freshly prepared mixture of acetonitrile and water (60:40, *v*/*v*) adjusted to pH 2.6 with formic acid, filtered using nylon membrane filters (0.45 µm) and degassed. The flow rate of the mobile phase was 1.0 mL/min, while the UV detection was set at 258 nm. All analyses were performed at room temperature 23 °C. Mefenamic acid was used as an I.S.

### 3.10. System Suitability

System suitability was determined before the sample analysis. Six solutions containing 15 µg/mL of CLB and 15 µg/mL of I.S. were prepared in methanol and injected onto the column. The similarity of peak area ratios of CLB to I.S. and similarity of retention times of both CLB and I.S. were taken into account.

### 3.11. Solutions for Calibration

The stock solution of CLB was prepared by dissolving the pure substance in methanol to obtain the concentration of 1.5 mg/mL. The stock solution of I.S. was prepared by dissolving the pure mefenamic acid in methanol to obtain the concentration of 1.0 mg/mL. The stock solution of CLB was diluted with methanol to obtain the working solutions at concentrations of 0.15 mg/mL (C1 solution), 0.075 mg/mL (C2 solution), 0.015 mg/mL (C3 solution), 0.0075 mg/mL (C4 solution), 0.0015 mg/mL (C5 solution) and 0.00075 mg/mL (C6 solution). 

### 3.12. Calibration

A 100 µL volume of the C6-C1 solutions of CLB and 650 µL of the solution of I.S. (1.0 mg/mL) were added to 250 µL of plasma, to obtain CLB concentrations of 0.075 µg/mL, 0.15 µg/mL, 0.75 µg/mL, 1.5 µg/mL, 7.5 µg/mL and 15 µg/mL. The samples were thoroughly vortexed and centrifuged at 5000 rpm at 23 °C for 5 min. The supernatants were injected onto the HPLC system through hydrophilic PTFE syringe filters (0.2 µm).

### 3.13. Precision and Accuracy

The stock solution of CLB was obtained in methanol at a concentration of 1.5 mg/mL and used for the QC samples at concentrations of 0.15 mg/mL (QC1 solution), 0.03 mg/mL (QC2 solution), 0.0015 mg/mL (QC3 solutions) and 0.00075 mg/mL (QC4 solution). These QC solutions were stored in the freezer at −20 °C and brought to room temperature for precision and accuracy, as well as recovery and stability studies.

The QC samples in plasma were prepared by adding 100 µL volumes of QC4-QC1 solutions to 250 µL of plasma and spiking with 650 µL of the I.S. working solution (1.0 mg/mL). In this way, CLB concentrations of 0.075 µg/mL (LLOQ level), 0.15 µg/mL (a low level), 3 µg/mL (a medium level), and 15 µg/mL (a high level) were obtained. The samples were thoroughly vortexed and centrifuged at 5000 rpm at 23 °C for 5 min. The supernatants were filtered through hydrophilic PTFE syringe filters and injected onto the HPLC system. The intra-batch precision and accuracy were determined by analyzing the QC samples five times on the same day. The inter-batch precision and accuracy were determined by analyzing the QC samples on two different days.

### 3.14. Recovery and Matrix Effect

The recovery of CLB from plasma was assessed in the QC samples at low (0.15 µg/mL), medium (3 µg/mL) and high (15 µg/mL) levels in five replicates, and calculated by comparing the determined concentrations with the nominal values. The matrix effect was evaluated by comparing the peak area ratios of CLB to I.S. which were obtained for the samples extracted from plasma, with the peak area ratios of the analytes at the same concentrations analyzed directly.

### 3.15. Chromatographic Conditions for VPA Assay

A GC chromatograph 7890A equipped with a 7692AALS autosampler coupled to a 7000A triple quadrupole mass spectrometer from Agilent (Agilent Technologies Deutschland GmbH, Waldbronn, Deutschland) was used. Separation was done using an HP-5MS UI column (30 m × 0.25 mm, 0.25 µm film thickness) from Agilent. Ionization was carried out by an electron impact (EI) ionization with SIM mode. Three SIM ions for VPA at *m*/*z* 159, 116, and 87, and for benzoic acid (I.S.) at *m*/*z* 136, 105 and 77 were selected for the quantitative determinations. The mass spectrometer was tuned manually using perfluorotributylamine from Sigma-Aldrich, with *m*/*z* 69 and 502. Helium was used as the carrier gas at a constant flow rate of 1 mL/min. The operating parameters used were, the injector temperature 250 °C, source temperature 280 °C, MS transfer line temperature 300 °C and quadrupole temperature 150 °C. The starting oven temperature was 80 °C (1.2 min held) and it increased at a rate of 25 °C/min to 250 °C (2 min held). Injections were done in the split mode with the split ratio 10:1. Standard electron impact conditions (70 eV) were used. For collision induced dissociation (CID), ultra-high purity nitrogen (1.5 mL/min) was used and 25 V as a collision cell voltage was applied. Helium gas (2.25 mL/min) was also used as a quenching gas to eliminate the meta stable helium species. The chromatographic data were analyzed by MassHunter Data Analysis Reporting B.07.01 software from Agilent.

### 3.16. Solutions for Calibration

The stock solution of VPA was prepared by dissolving the substance in methanol to obtain the concentration of 1.5 mg/mL. It was diluted with methanol to obtain working solutions at the following concentrations: 0.15 mg/mL (C1 solution), 0.075 mg/mL (C2 solution), 0.015 mg/mL (C3 solution), 0.0075 mg/mL (Q4 solution), 0.0015 (Q5 solution) and 0.00075 mg/mL (C6 solution). The stock solution of benzoic acid (I.S.) was prepared at a concentration of 1.0 mg/mL and diluted with methanol to obtain the working concentration of 0.05 mg/mL.

### 3.17. Derivatization and Calibration

To obtain VPA concentrations of 0.075 µg/mL, 0.15 µg/mL, 0.75 µg/mL, 1.50 µg/mL, 7.5 µg/mL and 15.0 µg/mL, aliquots of 100 µL of C6-C1 solutions and 650 µL of the I.S. working solution (0.05 mg/mL) were added to 250 µL aliquots of plasma. The samples were thoroughly vortexed and centrifuged at 5000 rpm at 4 °C for 5 min. Supernatant volumes of 200 µL were placed in chemically inert glass vials and subjected to derivatization and extraction procedures. Derivatization was carried out via simple esterification with methanol, according to the literature [32]. In short, volumes of 2 mL of sulphuric acid in methanol (10%, *v*/*v*) were added to each sample and thoroughly mixed. Then, the vials were heated at 60 °C for 30 min. After cooling to room temperature, 1 mL volumes of saturated sodium bicarbonate solution and 1 mL volumes of hexane were added to each sample for extraction. The mixtures were vortexed and centrifuged at 5000 rpm at 4 °C for 5 min. Then, aliquots of 500 µL of the upper (organic) layers were transferred to GC-MS vials, filled up with hexane to an equal volume of 1.5 mL and taken for GC-MS analysis.

### 3.18. Precision and Accuracy

The stock solution of VPA at a concentration of 1.5 mg/mL was prepared by independent weighing of the pure substance. Then, it was diluted to obtain the concentrations of 0.15 mg/mL (QC1 solution), 0.03 mg/mL (QC2 solution), 0.015 mg/mL (QC3 solution) and 0.0075 mg/mL (QC4 solution). These QC solutions were stored in the freezer at −20 °C and brought to room temperature before use.

The QC samples at concentrations of 0.075 µg/mL (LLOQ level), 0.15 µg/mL (a low level), 3.0 µg/mL (a medium level) and 15.0 µg/mL (a high level) were prepared by adding 100 µL volumes of QC4-QC1 solutions to 250 µL aliquots of plasma, and spiking with 650 µL of the I.S. working solution (0.05 mg/mL). The samples were mixed and centrifuged at 5000 rpm at 4 °C for 5 min. Then, 200 µL of the supernatants were subjected to derivatization and extraction procedures described above, and taken for GC-MS analysis.

### 3.19. Recovery and Matrix Effect

The recovery of VPA from plasma was assessed in the QC samples at low (0.15 µg/mL), medium (3 µg/mL) and high (15.0 µg/mL) levels in five replicates, and calculated by comparing the measured concentrations with the nominal values. The matrix effect was evaluated by comparing the peak area ratios of VPA to I.S. obtained after derivatization and extraction from plasma, with those obtained for the samples derivatized directly from methanol. In short, the solutions of VPA at three concentrations, i.e., 0.15 mg/mL, 0.03 mg/mL and 0.0015 mg/mL were prepared. Next, the 100 µL volumes of each VPA solution and 650 µL of I.S. working solution (0.05 mg/mL) were mixed and filled up with methanol to an equal volume of 1 mL. Then, 200 µL volumes were taken for esterification followed by heating at 60 °C, extraction with hexane and finally GC-MS analysis, as described above.

## 4. Conclusions

Accumulating data shows that VPA belongs to a group of epigenetic modifying agents and can effect many alterations in cancer cells, through modulation of multiple signaling pathways. Thus, VPA could be effective in some anticancer therapy, especially in combination with other anticancer drugs, e.g., CLB. Our in vitro study (sent for publication, under review) confirmed potent anticancer activity of VPA in CLL cells, especially due to the enhancement of CLB activity when used in combination. Based on these results, we can conclude that the combined treatment with VPA and CLB might be a new alternative in the therapy of CLL. Thus, two simple quantitative HPLC-UV and GC-MS methods were elaborated and validated for further studies of CLB and VPA in cultured cells and plasma, and to examine pharmacological aspects of such a combined therapy. As was stated above, in the literature there is no method for the simultaneous determination of CLB and VPA in biological materials. What is more, there are a limited number of procedures for the determination of CLB, as well as a limited number of simple methods for the determination of VPA. As a consequence, their determination in plasma is an ongoing challenge for researchers. On the one hand, it was not possible for us to develop one simple method for the simultaneous determination of CLB and VPA with the required sensitivity, accuracy and precision, mainly due to large differences in their physico-chemical properties. On the other hand, the presented methods allow the determination of CLB and VPA in the same plasma samples, without any mutual interference. As far as the required sensitivity, linearity, precision, accuracy, matrix effect, carryover and stability are concerned, they could be good alternatives to previously described HPLC or LC-MS methods. In addition, we think that the GS-MS method as such, should be promoted on a large scale in medical institutions at all levels as a suitable method for bioanalytical studies. What is more, both methods could be easily adopted for monitoring of CLB and VPA in a variety of in vitro as well as ex vivo scenarios, e.g., cell cultures.

## Figures and Tables

**Table 1:** Parameters of HPLC-UV method for the determination of chlorambucil (CLB) in plasma (*n* = 6).

Parameter	Values
Retention time for CLB (mean ± SD) [min]	2.114 ± 0.019
Internal standard (I.S.)	mefenamic acid
Retention time for I.S. (mean ± SD) [min]	3.245 ± 0.026
Resolution (between CLB and I.S.)	4.2
System suitability (RSD for CLB/I.S. peak areas ratio) [%]	3.58
Linearity range [µg/mL]	0.075-15
Slope	0.15501
SD of the slope	0.00142
Intercept	0.00244
SD of the intercept	0.00123
r^2^	0.9996
The limit of detection (LOD) [µg/mL]	0.024
The lower limit of quantification (LLOQ) [µg/mL]	0.075

SD = standard deviation.

**Table 2:** Intra-batch and inter-batch precision and accuracy of CLB assay (*n* = 5).

Spiked conc. [µg/mL]	Intra-Batch Precision and Accuracy			Inter-Batch Precision and Accuracy		
	Measured conc. Mean ± SD [µg/mL]	CV [%]	Accuracy [Recovery]	Measured conc. Mean ± SD [µg/mL]	CV [%]	Accuracy [Recovery]
LLOQ	0.0706 ± 0.0064	9.09	94.13	0.0715 ± 0.0071	9.82	95.33
QC 0.15	0.1428 ± 0.0135	9.46	95.21	0.1464 ± 0.0099	6.81	97.60
QC 3.0	3.0337 ± 0.1089	3.59	101.12	2.8991 ± 0.1166	4.02	96.64
QC 15.0	14.5761 ± 0.3694	2.53	97.14	14.2586 ± 0.4651	3.26	95.06

Conc. = concentration; CV = coefficient of variation; QC = Quality Control.

**Table 3:** Recovery and matrix effect of CLB in plasma (*n* = 3).

Chemical Standards [µg/mL]	Measured Conc. Mean ± SD [µg/mL]	Spiked Conc. in Plasma [µg/mL]	Measured Conc. Mean ± SD [µg/mL]	Matrix Effect [%]
0.15	0.1497 ± 0.0071	QC 0.15	0.1428 ± 0.0135	95.45 ± 8.04
3.0	3.0471 ± 0.1315	QC 3.0	3.0337 ± 0.1089	100.33 ± 6.78
15.0	15.1031 ± 0.2509	QC 15.0	14.5761 ± 0.3694	96.55 ± 3.49

**Table 4:** Stability data of CLB in plasma (*n* = 3).

Storage Conditions	Spiked Conc. [µg/mL]	Measured Conc. Mean ± SD [µg/mL]	RSD [%]	RE [%]
Short-term	QC 0.15	0.1472 ± 0.0054	3.67	1.87
	QC 15.0	14.5576 ± 0.3761	2.58	2.95
Long-term	QC 0.15	0.1403 ± 0.0082	5.84	2.39
	QC 15.0	14.3432 ± 0.3542	2.47	4.38
Freeze-thaw	QC 0.15	0.1405 ± 0.0089	6.33	6.33
	QC 15.0	14.2486 ± 0.3465	2.43	5.01
Post-preparative	QC 0.15	0.1423 ± 0.0071	4.99	5.13
	QC 15.0	14.1032 ± 0.2741	1.94	5.98

RE = relative error; RSD = relative standard deviation.

**Table 5:** Parameters of GC-MS method for the determination of valproic acid (VPA) in plasma (*n* = 6).

Parameter	Values
Acquisition time for VPA (mean ± SD) [min]	3.076 ± 0.009
SIM ions for VPA (*m*/*z*)	159, 116, 87
I.S.	benzoic acid
Acquisition time for I.S. (mean ± SD) [min]	3.587 ± 0.008
SIM ions for I.S. (*m*/*z*)	136, 105, 77
Linearity range [µg/mL]	0.075–15.0
Slope	0.32003
SD of the slope	0.01209
Intercept	−0.02029
SD of the intercept	0.00279
r^2^	0.9987
LOD [µg/mL]	0.026
LLOQ [µg/mL]	0.075

**Table 6:** Intra-batch and inter-batch precision and accuracy of VPA assay (*n* = 5).

Spiked Conc. [µg/mL]	Intra-Batch Precision and Accuracy			Inter-Batch Precision and Accuracy		
	Measured Conc. Mean ± SD [µg/mL]	CV [%]	Accuracy [%]	Measured Conc. Mean ± SD [µg/mL]	CV [%]	Accuracy [%]
LLOQ	0.0706 ± 0.0041	5.78	94.13	0.07122 ± 0.0048	6.69	94.96
QC 0.15	0.1426 ± 0.0042	2.96	95.07	0.1436 ± 0.0051	3.58	95.73
QC 3.0	3.2735 ± 0.1196	3.65	109.12	3.2427 ± 0.1863	5.75	108.09
QC 15.0	15.3429 ± 0.2282	1.45	102.29	15.3954 ± 0.3364	2.19	102.64

CV = coefficient of variation.

**Table 7:** Recovery and matrix effect of VPA in plasma (*n* = 3).

Chemical Standards [µg/mL]	Measured Conc. Mean ± SD [µg/mL]	Spiked Conc. in Plasma [µg/mL]	Measured Conc. Mean ± SD [µg/mL]	Matrix Effect [%]
0.15	0.1441 ± 0.0014	QC 0.15	0.1427 ± 0.0042	99.03 ± 3.31
1.5	1.5991 ± 0.0979	QC 1.5	1.5735 ± 0.1196	98.39 ± 2.51
15.0	15.1453 ± 0.1891	QC 15.0	15.3429 ± 0.2282	101.31 ± 2.73

**Table 8:** Stability data of VPA in plasma (*n* = 3).

Storage Conditions	Spiked Conc. [µg/mL]	Measured Conc. Mean ± SD [µg/mL]	RSD [%]	RE [%]
Short-term	QC 0.15	0.1453 ± 0.0067	4.61	3.13
	QC 15.0	14.2586 ± 0.4751	3.33	4.94
Long-term	QC 0.15	0.1395 ± 0.0111	7.96	7.01
	QC 15.0	14.1486 ± 0.4398	3.11	5.68
Freeze-thaw	QC 0.15	0.1446 ± 0.0125	8.64	3.60
	QC 15.0	13.0295 ± 0.3325	2.55	3.14
Post-preparative	QC 0.15	0.1436 ± 0.0116	8.08	4.27
	QC 15.0	13.8795 ± 0.3125	2.25	7.47

RE = relative error.

## Data Availability

The data presented in this study are available on request from the corresponding author.

## Supplementary Materials

The following are available online, Figure S1: Representative LC chromatogram obtained for VPA at concentration of 30 µg/mL, using UV detection at 210 nm; Figure S2. Representative GC chromatograms of plasma samples spiked with VPA and CLB (both at concentration of 15 µg/mL) at two different scales (a,b) and the MS-SIM mode spectrum of CLB (c); Figure S3. HPLC chromatograms of: (a) CLB (30 µg/mL) with the internal standard (I.S.), (b) CLB (30 µg/mL), I.S. and VPA (30 µg/mL) and (c) VPA (30 µg/mL) and I.S. (VPA was not detected because of a lack of respective chromophores and absorptivity at 258 nm).

## Sample Availability

Samples of chlorambucil and valproic acid are not available from the authors.
