# Supplementary material for: HPLC-UV and GC-MS Methods for Determination of Chlorambucil and Valproic Acid in Plasma for Further Exploring a New Combined Therapy of Chronic Lymphocytic Leukemia
Source: Molecules. 2021 May 13;26(10):2903. doi: 10.3390/molecules26102903 (PMC8153269; doi:10.3390/molecules26102903)
Supplement: Supplementary file 1 [file molecules-26-02903-s001.zip › molecules-1196585-supplementary/Figure S2.pdf]

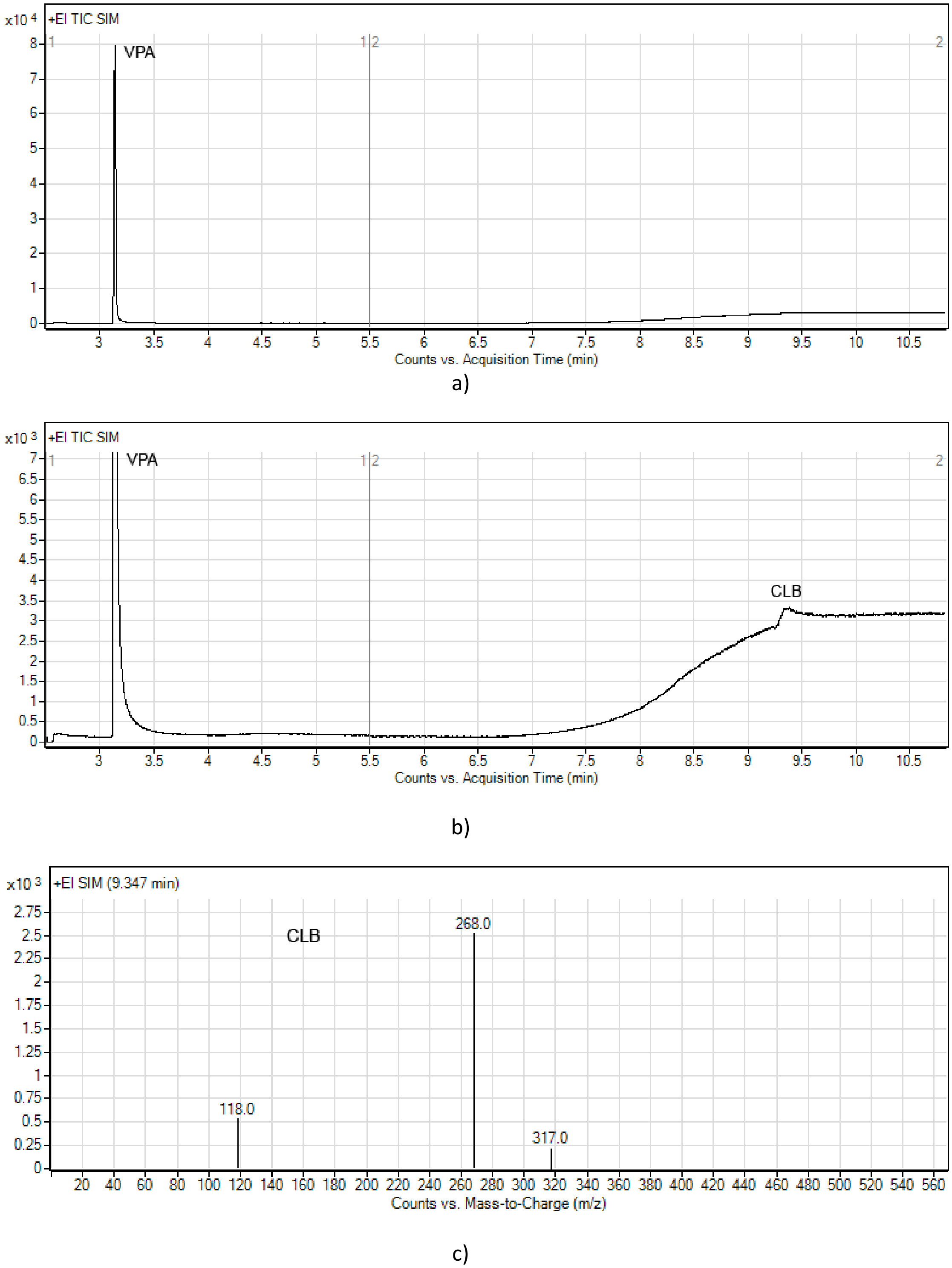

Figure S2. Representative GC chromatograms of plasma samples spiked with VPA and CLB (both at concentration of 15 µg/mL) at two different scales (a,b) and the MS-SIM mode spectrum of CLB (c).
