# Supplementary material for: HPLC-UV and GC-MS Methods for Determination of Chlorambucil and Valproic Acid in Plasma for Further Exploring a New Combined Therapy of Chronic Lymphocytic Leukemia
Source: Molecules. 2021 May 13;26(10):2903. doi: 10.3390/molecules26102903 (PMC8153269; doi:10.3390/molecules26102903)
Supplement: Supplementary file 1 [file molecules-26-02903-s001.zip › molecules-1196585-supplementary/Figure S3.pdf]

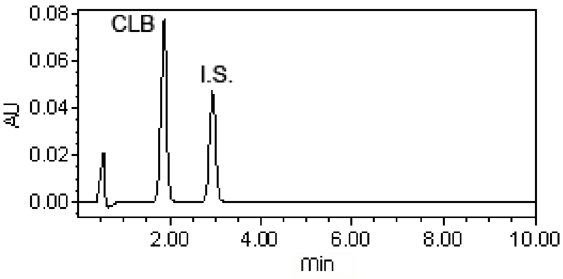

a)

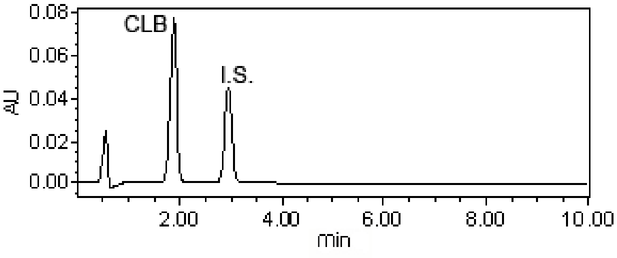

b)

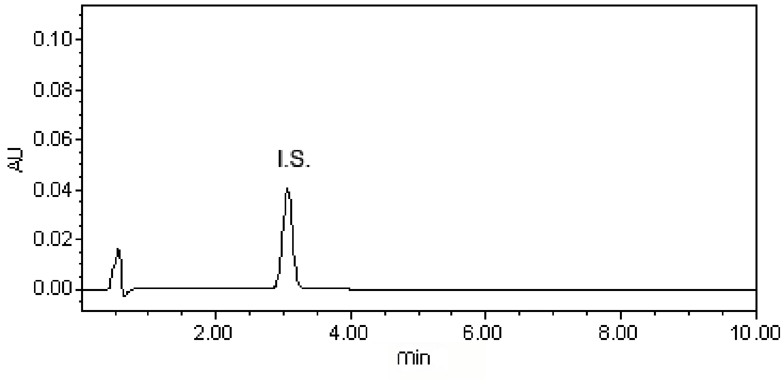

c)

Figure S3. HPLC chromatograms of: a) CLB (30 μg/mL) with the internal standard (I.S.), b) CLB (30 μg/mL), I.S. and VPA (30 μg/mL) and c) VPA (30 μg/mL) and I.S. (VPA was not detected because of a lack of respective chromophores and absorptivity at 258 nm).
